# Dysnatremia at ICU admission and functional outcome of cardiac arrest: insights from four randomised controlled trials

**DOI:** 10.1186/s13054-023-04715-z

**Published:** 2023-12-01

**Authors:** Jean Baptiste Lascarrou, Cyrielle Ermel, Alain Cariou, Timo Laitio, Hans Kirkegaard, Eldar Søreide, Anders M. Grejs, Matti Reinikainen, Gwenhael Colin, Fabio Silvio Taccone, Amélie Le Gouge, Markus B. Skrifvars

**Affiliations:** 1 Nantes Université, CHU Nantes, Movement – Interactions – Performance, MIP, UR 4334, 44000 Nantes, France; 2 Médecine Intensive Reanimation, University Hospital Centre, Nantes, France; 3 AfterROSC Network, Nantes, France; 4 Université de Paris Cité, INSERM, Paris Cardiovascular Research Centre, Paris, France; 5 Médecine Intensive Reanimation, AP-HP, CHU Cochin, Paris, France; 6 Division of Perioperative Services, Intensive Care Medicine and Pain Management, Turku University Hospital, University of Turku, Turku, Finland; 7 Research Centre for Emergency Medicine and Anaesthesiology and Intensive Care, Aarhus University Hospital and Department of Clinical Medicine, Aarhus University, Aarhus, Denmark; 8 Intensive Care Unit, Department of Anaesthesiology, Stavanger University Hospital and Faculty of Health Sciences, University of Stavanger, Stavanger, Norway; 9 Department of Intensive Care Medicine, Aarhus University Hospital, Aarhus, Denmark; 10 Department of Clinical Medicine, Aarhus University, Aarhus, Denmark; 11 Department of Anaesthesiology and Intensive Care, Kuopio University Hospital, Kuopio, Finland; 12 University of Eastern Finland, Kuopio, Finland; 13 Médecine Intensive Reanimation, CHD Vendee, La Roche Sur Yon, France; 14 Department of Intensive Care, Hôpital Universitaire de Bruxelles (HUB), Université Libre de Bruxelles (ULB), Brussels, Belgium; 15 INSERM CIC 1415, CHRU Tours, Tours, France; 16 Department of Emergency Care and Services, Helsinki University Hospital and University of Helsinki, Helsinki, Finland; 17 Service de Médecine Intensive Reanimation, CHU Nantes, 30 Boulevard Jean Monet, 44093 Nantes Cedex 9, France

**Keywords:** Cardiac arrest, Natremia, Functional outcome, Osmolality, Survival

## Abstract

**Purpose:**

To evaluate the potential association between early dysnatremia and 6-month functional outcome after cardiac arrest.

**Methods:**

We pooled data from four randomised clinical trials in post-cardiac-arrest patients admitted to the ICU with coma after stable return of spontaneous circulation (ROSC). Admission natremia was categorised as normal (135–145 mmol/L), low, or high. We analysed associations between natremia category and Cerebral Performance Category (CPC) 1 or 2 at 6 months, with and without adjustment on the modified Cardiac Arrest Hospital Prognosis Score (mCAHP).

**Results:**

We included 1163 patients (581 from HYPERION, 352 from TTH48, 120 from COMACARE, and 110 from Xe-HYPOTHECA) with a mean age of 63 ± 13 years and a predominance of males (72.5%). A cardiac cause was identified in 63.6% of cases. Median time from collapse to ROSC was 20 [15–29] minutes. Overall, mean natremia on ICU admission was 137.5 ± 4.7 mmol/L; 211 (18.6%) and 31 (2.7%) patients had hyponatremia and hypernatremia, respectively. By univariate analysis, CPC 1 or 2 at 6 months was significantly less common in the group with hyponatremia (50/211 [24%] vs. 363/893 [41%]; *P* = 0.001); the mCAHP-adjusted odds ratio was 0.45 (95%CI 0.26–0.79, *p* = 0.005). The number of patients with hypernatremia was too small for a meaningful multivariable analysis.

**Conclusions:**

Early hyponatremia was common in patients with ROSC after cardiac arrest and was associated with a poorer 6-month functional outcome. The mechanisms underlying this association remain to be elucidated in order to determine whether interventions targeting hyponatremia are worth investigating.

*Registration* ClinicalTrial.gov, NCT01994772, November 2013, 21.

**Supplementary Information:**

The online version contains supplementary material available at 10.1186/s13054-023-04715-z.

## Introduction

Secondary insults can substantially worsen the outcomes of patients admitted to the intensive care unit (ICU) with brain injury. Studies done over the last 50 years have demonstrated strong associations linking glycaemic control, body temperature [1], oxygenation, and disturbances in metabolites and electrolytes to outcomes of brain injured patients [2]. Several of these factors have been extensively studied in patients admitted to the ICU with post-cardiac-arrest brain injury. Notably, an observational study showing that fever was associated with poorer outcomes after cardiac arrest [3] prompted several randomised controlled trials (RCTs) of therapeutic hypothermia [4–6]. Also, guidelines about fever prevention and patient selection for neuroprotective interventions after cardiac arrest have been developed [6, 7].

Brain ischaemia and brain oedema are two major components of secondary brain injuries. As a major determinant of free-water shifts and intracellular hydration, natremia strongly influences brain volume. About a third of ICU patients overall have abnormal serum sodium levels at admission [8]. Serum sodium abnormalities are associated with neurological manifestations ranging from impaired consciousness to osmotic demyelination syndrome [9]. Even though cardiac arrest is a common life- and function-threatening event, with over 300000 cases annually in Europe [10], data on potential links between dysnatremia and cardiac-arrest outcomes are scant. A study comparing intraosseous, arterial, and central venous samples demonstrated dynamic natremia changes during and early after cardiac arrest [11]. In a study of 80 patients, natremia increased from 137 (104–143) mmol/L during cardiac arrest to 140 (109–142) immediately after ROSC [12]. Collecting further information on the relations between natremia and outcomes of cardiac arrest might help to determine whether interventions targeting dysnatremia hold promise for improving outcomes.

The primary objective of this post hoc study was to use data from four published RCTs to look for associations linking dysnatremia early in the ICU stay to functional outcome on day 180 after cardiac arrest. The secondary objective was to determine whether dysnatremia was associated with 6-month survival.

## Materials and methods

### Study design and study settings

We performed a post hoc analysis of data collected prospectively in four published RCTs of neuroprotective interventions after cardiac arrest: HYPERION (hypothermia at 33 °C [5]), COMACARE (modified Mean Arterial Pressure, modified arterial CO_2_ and O_2_ levels [13]), TTH48 (hypothermia for 24 h vs. 48 h [14]), and Xe-HYPOTHECA (inhaled xenon [15]). Only HYPERION found a significant improvement in neurological outcome with the trial intervention. These four trials were chosen because they enrolled similar populations of patients with sustained ROSC after cardiac arrest and ICU admission with coma. Also, the process of care and the inclusion window were similar in the four trials.

The four trials enrolled 1172 ICU patients in all between August 2009 and January 2018. For our study, we included those patients admitted to the ICU alive and for whom laboratory results were available then evaluated their identified data.

### Ethics

The study was approved by the ethics committee of the French Intensive Care Society (FICS/SRLF 23/036) and was conducted in compliance with the latest version of the Declaration of Helsinki and good clinical practice guidelines. The protocol of each of the four RCTs was approved by the appropriate ethics committees.

### Study population

All patients admitted to the participating centres during each trial period were screened for eligibility. Inclusion criteria were age 18 years or older (in the TTH48 trial up to 80 years) and ICU admission after out-of-hospital cardiac arrest (OHCA), or in-hospital cardiac arrest in HYPERION, followed by the return of spontaneous circulation (ROSC) with persistent coma (defined as a Glasgow Coma Scale [GCS] score ≤ 8). We did not include data from patients who withdrew consent after initial inclusion, had randomisation errors, or did not receive the allocated intervention.

### Definition of metabolic disorders

We defined serum sodium levels of 135–145 mmol/L as normal natremia [16], < 135 mmol/L as hyponatremia, and > 145 mmol/L as hypernatremia. We chose to divide the natremia values into three categories to facilitate interpretation by clinicians and to increase relevance to any possible future trials comparing targeted normonatremia to dysnatremia. We considered the first serum sodium level obtained after ICU admission.

Osmolality was computed as follows: (natremia·2) + glycaemia [17]. Osmolality < 275 mOsm/kg defined hypo-osmolality and ≥ 295 mOsm/kg hyperosmolality.

### Data collection

For each patient in each RCT, a dedicated study nurse or investigator at each participating centre collected the baseline clinical data and comorbidities; characteristics of the cardiac arrest and resuscitation; clinical and laboratory features at ICU admission; treatments delivered in the ICU; ICU length of stay; invasive mechanical ventilation duration; and vital and functional status at ICU discharge and hospital discharge and at long-term follow-up.

### Outcome measures

For the present study, the primary outcome was the day-180 Cerebral Performance Category (CPC) [18]. The day-180 CPC was the primary outcome in the TTH48 trial and a secondary outcome in the COMACARE and Xe-HYPOTHECA trials; in HYPERION, the primary outcome was the CPC on day 90, and the value for day 180 was impute using the last-observation-carried-forward method. A poor outcome was defined as a CPC of 3 (moderately severe disability), 4 (severe disability), or 5 (dead), as recommended in guidelines [19].

The secondary outcome for the present study was 6-month survival.

### Statistical analysis

Given the exploratory nature of our analysis, we did not perform a formal sample size estimation. Categorical variables were described as proportions and continuous variables as mean ± SD if normally distributed and as median [interquartile range] otherwise.

To look for associations linking dysnatremia to day-180 CPC, we performed binary logistic mixed regression analyses with a random effect on the trial. Multivariable analysis adjusted on the modified Cardiac Arrest Hospital Prognosis (mCAHP) score [20] was pre-planned. The mCAHP was introduced in the model as a continuous variable. This tool is a summary statistic built to predict brain damage early after cardiac arrest with ROSC, thereby enabling comparisons of different populations of patients with cardiac arrest [20–22]. The seven variables are age, setting of cardiac arrest (public place/home), initial rhythm (shockable/not shockable), time from collapse to CPR initiation, time from CPR initiation to ROSC, blood pH at ICU admission, and epinephrine dose (0/1–2 mg/ ≥ 3 mg).

A sensitivity analysis was performed by considering three groups, defined by hypo-osmolality, normal osmolality, and hyperosmolality, respectively, then performing univariate analyses to look for associations with day-180 CPC before and after adjustment on the mCAHP. Finally, all analyses were repeated with day-180 survival as the endpoint.

The CPC in the HYPERION trial [5] was available only until day 90, instead of day 180 in the three other trials. For HYPERION, we used the last-observation-carried-forward method to perform imputation for the day-180 values of the CPC and mortality. However, since this method assumes stability of values over time (here, from day 90 to day 180), we also performed a sensitivity analysis using only the data from the three other trials.

All tests were two-sided, and *p* values < 0.05 were considered significant. The analyses were performed using SAS version 9.4 (SAS Institute, Cary, NC) and R version 3.3.1.

## Results

### Baseline characteristics

**Fig. 1:**
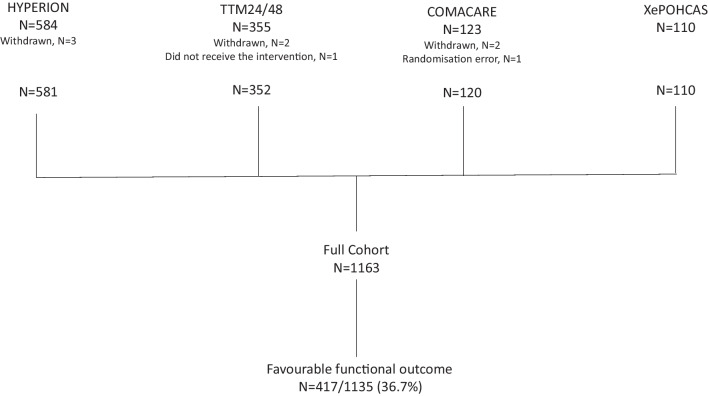
Study flowchart

Our analyses included 1163 patients (581 from HYPERION, 352 from TTH48, 120 from COMACARE, and 110 from Xe-HYPOTHECA) (Fig. 1). Data on whether the outcome was favourable were missing for 28 patients. Table 1 reports the main baseline features. Males predominated and two-thirds of patients had a cardiac cause of arrest. Mean age was 62.9 ± 13.2 years.

**Table 1:** Baseline characteristics of the populations in each of the four trials

	**All** **(n = 1163)**	**Hyperion** **(n = 581)**	**TTM24/48** **(n = 352)**	**Comacare** **(n = 120)**	**Xenon** **(n = 110)**
Male, n (%)	843 (72.5)	373 (64.2)	292 (82.9)	98 (81.7)	80 (72.7)
Age, years, mean ± SD	62.9 ± 13.2	65.7 ± 13.7	60.3 ± 11.8	59.6 ± 13.0	59.9 ± 11.2
Height, cm, mean ± SD	172.5 ± 10.2	168.1 ± 9.0	177.7 ± 8.2	176.4 ± 8.9	173.4 ± 13.1
Weight, kg, mean ± SD	82.3 ± 18.9	79.3 ± 20.1	85.8 ± 16.2	84.4 ± 16.7	84.8 ± 20.6
Body Mass Index, kg·m^2^, mean ± SD	28.0 ± 11.2	28.2 ± 7.0	27.2 ± 4.6	27.1 ± 5.0	30.5 ± 30.6
Glasgow Coma Scale score, median [Q1–Q3]	3.0 [3.0–3.0]	3.0 [3.0–3.0]	-	3.0 [3.0–3.0]	3.0 [3.0–3.0]
Cardiac cause of arrest, n (%)	740 (63.6)	158 (27.2)	352 (100.0)	120 (100.0)	110(100.0)
Shockable rhythm, n (%)	542 (46.6)	0 (0.0)	312 (88.6)	120 (100.0)	110 (100.0)
Chronic heart disease, n (%)	522 (44.9)	342 (58.9)	18 (5.1)	86 (71.7)	76 (69.1)
Chronic pulmonary disease, n (%)	250 (21.5)	204 (35.1)	24 (6.8)	8 (6.7)	14 (12.7)
Bystander witnessed cardiac arrest, n (%)	1100/1162 (94.7)	547/580 (94.3)	323 (91.8)	120 (100.0)	110 (100.0)
Bystander performed CPR, n (%)	875/1161 (75.4)	407/579 (74.4)	293 (83.2)	98 (81.7)	77 (70.0)
*Location at cardiac arrest*					
Place of residence, n (%)	601 (51.7)	295 (50.8)	192 (54.5)	60 (50.0)	54 (49.1)
Public place, n (%)	402 (34.6)	127 (21.9)	160 (45.5)	60 (50.0)	55 (50.0)
Hospital, n (%)	160 (13.8)	159 (27.4)	0 (0.0)	0 (0.0)	1 (0.9)
Body temperature, °C, mean ± SD	35.2 ± 1.4	35.4 ± 1.5	34.9 ± 1.1	-	35.1 ± 1.2
SAPSII, median [Q1–Q3]	71.0 [58.0–82.0]	73.5 [63.0–84.5]	-	-	52.0 [42.0–60.0]
No-flow duration, minutes, median [Q1–Q3]	0.0 [0.0–5.0]	2.0 [0.0–5.0]	0.0 [0.0–1.0]	-	0.0 [0.0–5.0]
Low-flow duration, minutes, median [Q1–Q3]	18.0 [12.0–26.0]	16.0 [10.0–25.0]	20.0 [15.0–27.0]	-	19.0 [14.0–26.0]
Time to ROSC, median [Q1–Q3]	20.0 [15.0–29.0]	20.0 [12.0–30.0]	21.0 [15.0–28.0]	21.0 [16.0–26.0]	21.0 [16.0–27.0]
Epinephrine injection performed, n (%)	828/1043 (79.4)	535 (92.1)	222 (63.1)	-	71 (64.5)
Epinephrine dose, mg, median [Q1–Q3]	2.0 [1.0–4.0]	3.0 [2.0–5.0]	0.0 [0.0–2.0]	-	1.0 [0.0–2.2]
Amiodarone injection, n (%)	251/1043 (24.1)	53 (9.1)	145 (41.2)	-	53 (48.2)
Bicarbonate injection n (%)	59/1043 (5.7)	49 (8.4)	6 (1.7)	-	4 (3.6)
Amines, n (%)	467/811 (57.6)	339 (58.3)	-	58 (48.3)	70 (63.6)
Successful angioplasty, n (%)	265/1161 (22.8)	40 (6.9)	145/350 (41.4)	46 (38.3)	34 (30.9)
Coronarography, n (%)	600 (51.6)	148 (25.5)	291 (82.7)	70 (58.3)	91 (82.7)
Admission pH, median [Q1–Q3]	7.3 [7.2–7.3]	7.2 [7.1–7.3]	7.3 [7.2–7.3]	7.3 [7.3–7.4]	7.3 [7.2–7.4]
Lactates, mmol/L, median [Q1–Q3]	3.6 [1.9–7.0]	6.1 [3.2–9.7]	2.6 [1.4–5.0]	2.1 [1.3–3.4]	2.4 [1.4–3.6]
Serum creatinine, µmol/L, median [Q1–Q3]	101.0 [80.0–133.0]	112.0 [85.0–153.5]	99.0 [82.0–121.0]	85.0 [73.0–103.0]	88.0 [76.0–111.0]
Blood glucose, mmol/L, mean ± SD	12.3 ± 6.8	13.1 ± 6.3	-	10.7 ± 3.9	10.7 ± 10.1
PaCO_2_, mmHg, mean ± SD	45.8 ± 14.0	47.7 ± 17.5	45.7 ± 9.5	40.6 ± 8.1	42.4 ± 8.8
PaO_2_, mmHg, mean ± SD	170.5 ± 115.3	195.6 ± 136.9	145.4 ± 82.1	129.0 ± 56.0	170.3 ± 106.2
NSE on day 2, ng/mL, median [Q1–Q3]	24.0 [17.8–36.8]	47.0 [18.8–172.3]	-	21.2 [13.8–33.1]	23.9 [19.6–30.0]
NSE on day 3, ng/mL, median [Q1–Q3]	21.3 [14.9–45.0]	43.3 [17.3–199.7]	-	16.7 [12.2–26.0]	21.8 [17.0–34.3]
Natremia, mmol/L, mean ± SD	137.5 ± 4.7	137.5 ± 5.7	137.7 ± 3.7	136.9 ± 3.2	137.7 ± 3.4
Normonatremia, n (%)	893 (78.7)	408 (72.9)	295 (85.5)	95 (79.2)	95 (86.4)
Hyponatremia, n (%)	211 (18.6)	125 (22.3)	46 (13.3)	25 (20.8)	15 (13.6)
Hypernatremia, n (%)	31 (2.7)	27 (4.8)	4 (1.2)	0 (0.0)	0 (0.0)
Potassium, mmol/L, mean ± SD	4.3 ± 1.0	4.5 ± 1.1	4.1 ± 0.7	4.2 ± 0.8	4.1 ± 0.7
Urea, mmol/L, mean ± SD	8.7 ± 7.4	9.7 ± 6.9	8.2 ± 9.5	6.5 ± 2.5	7.3 ± 4.3
Normo-osmolality, n (%)	490 (67.1)	299 (59.0)	-	96 (84.2)	95 (87.2)
Hypo-osmolality, n (%)	80 (10.9)	65 (12.8)	-	9 (7.9)	6 (5.5)
Hyperosmolality, n (%)	160 (21.9)	143 (28.2)	-	9 (7.9)	8 (7.3)
mCAHP score, median [Q1–Q3]	90.8 [73.1–106.5]	100.9 [88.8–112.5]	70.7 [54.5–84.2]	-	71.2 [57.6–85.2]

CPR: cardiopulmonary resuscitation; SAPSII: Simplified Acute Physiology Score version II; ROSC: return of spontaneous circulation; PaO_2_: partial pressure of oxygen in arterial blood; PaCO_2_: partial pressure of carbon dioxide in arterial blood; NSE: neuron-specific enolase; mCAHP: modified Cardiac Arrest Hospital Prognosis

Overall, the first recorded mean natremia value was 137.5 ± 4.7 mmol/L. Table 2 reports the main features in the groups with normal natremia (n = 893, 78.7%), hyponatremia (n = 211, 18.6%), and hypernatremia (n = 31, 2.7%). Natremia was not available for 27 patients.

**Table 2:** Baseline characteristics according to natremia category

	Normonatremia (n = 893)	Hyponatremia (n = 211)	**Hypernatemia** **(n = 31)**
Males, n (%)	651 (72.9)	156 (73.9)	19 (61.3)
Age, years, mean ± SD	62.9 ± 13.2	63.2 ± 12.2	60.1 ± 18.3
Height, cm, mean ± SD	172.9 ± 10.5	172.1 ± 8.8	168.4 ± 10.2
Weight, Kg, mean ± SD	83.3 ± 18.9	80.0 ± 18.2	75.2 ± 22.9
Body Mass Index, Kg·m^2^, mean ± SD	28.2 ± 12.3	27.3 ± 6.0	27.3 ± 7.9
Glasgow Coma Scale score at enrolment, median [Q1–Q3]	3.0 [3.0–3.0]	3.0 [3.0–3.0]	3.0 [3.0–3.0]
Cardiac cause of arrest, n (%)	596 (66.7)	121 (57.3)	8 (25.8)
Shockable rhythm, n (%)	452 (50.6)	80 (37.9)	4 (12.9)
Chronic heart disease, n (%)	390 (43.7)	112 (53.1)	12 (38.7)
Chronic pulmonary disease, n (%)	168 (18.8)	70 (33.2)	7 (22.6)
Bystander witnessed cardiac arrest, n (%)	848 (95.0)	195 (92.9)	29 (93.5)
Bystander performed CPR, n (%)	674 (75.6)	149 (71.0)	26 (83.9)
*Location at cardiac arrest*			
Place of residence, n (%)	478 (53.5)	102 (48.3)	8 (25.8)
Public place, n (%)	315 (35.3)	68 (32.2)	10 (32.3)
Hospital, n (%)	100 (11.2)	41 (19.4)	13 (41.9)
Body temperature, °C, mean ± SD	35.2 ± 1.3	35.2 ± 1.5	35.2 ± 1.7
SAPSII, mean ± SD	69.0 ± 17.7	72.2 ± 17.5	78.0 ± 14.0
No-flow duration minutes, median [Q1–Q3]	0.0 [0.0–5.0]	0.0 [0.0–5.0]	0.5 [0.0–5.0]
Low-flow duration minutes, median [Q1–Q3]	18.0 [12.0–26.0]	18.0 [10.0–25.0]	21.0 [10.0–32.0]
Time to ROSC, minutes, median [Q1–Q3]	20.0 [15.0–28.0]	21.0 [15.0–30.0]	20.5 [11.0–39.0]
Epinephrine injection performed, n (%)	618 (77.4)	157 (84.4)	29 (93.5)
Epinephrine dose, mg, median [Q1–Q3]	2.0 [0.0–4.0]	2.0 [1.0–4.0]	4.0 [2.0–6.0]
Amiodarone injection, n (%)	216 (27.1)	32 (17.2)	2 (6.5)
Bicarbonate injection, n (%)	40 (5.0)	9 (4.8)	8 (25.8)
Vasopressor at inclusion n (%)	336 (56.2)	97 (58.8)	22 (81.5)
ST-elevation myocardial infarction n (%)	365 (41.7)	66 (32.2)	5 (17.9)
Coronary angiography, n (%)	480 (53.8)	104 (49.3)	4 (12.9)
Successful angioplasty, n (%)	215 (24.1)	42 (19.9)	2 (6.5)
Admission pH, median [Q1–Q3]	7.3 [7.2–7.3]	7.3 [7.2–7.3]	7.2 [7.0–7.3]
Lactates, mmol/L, median [Q1–Q3]	3.4 [1.8–6.7]	3.9 [1.9–7.2]	9.4 [4.8–14.6]
Serum creatinine, µmol/L, median [Q1–Q3]	100.0 [80.0–129.0]	103.0 [78.0–143.0]	137.0 [86.0–183.0]
Blood glucose, mmol/L, mean ± SD	11.9 ± 5.4	13.9 ± 10.2	11.9 ± 8.0
PaCO_2_, mmHg, mean ± SD	45.8 ± 13.1	45.1 ± 16.9	50.1 ± 19.0
PaO_2_, mmHg, mean ± SD	169.3 ± 113.8	171.7 ± 117.2	202.4 ± 140.8
NSE on day 2, ng/mL, median [Q1–Q3]	23.9 [18.0–35.8]	24.0 [17.4–35.0]	63.4 [9.0–90.2]
NSE on day 3, ng/mL, median [Q1–Q3]	20.9 [14.7–44.0]	21.7 [15.4–48.5]	26.9 [5.9–33.9]
Potassium, mmol/L, mean ± SD	4.2 ± 0.9	4.7 ± 1.1	4.7 ± 1.5
Urea, mmol/L, mean ± SD	8.4 ± 6.1	9.7 ± 10.9	11.3 ± 8.9
Normo-osmolality, n (%)	414 (75.5)	75 (48.4)	1 (3.7)
Hypo-osmolality, n (%)	2 (0.4)	78 (50.3)	0 (0.0)
Hyperosmolality, n (%)	132 (24.1)	2 (1.3)	26 (96.3)
mCAHP score, median [Q1–Q3]	90.6 [71.2–106.8]	91.4 [79.0–105.5]	96.0 [84.3–109.3]

CPR: cardiopulmonary resuscitation; SAPSII: Simplified Acute Physiology Score version II; ROSC: return of spontaneous circulation; PaO_2_: partial pressure of oxygen in arterial blood; PaCO_2_: partial pressure of carbon dioxide in arterial blood; NSE: neuron-specific enolase; mCAHP: modified Cardiac Arrest Hospital Prognosis

### Associations with day-180 cerebral performance category

The day-180 CPC was 1 or 2 (defining a favourable outcome) in 46/581 (7.9%), 225/352 (63.9%), 78/120 (65.0%), and 72/110 (65.5%) patients in the HYPERION, TTH48, COMACARE, and Xe-HYPOTHECA trials, respectively (Table 3). The AUROC for the mCAHP is shown in Additional file 2: eFigure 1.

**Table 3:** Favourable neurological prognosis according to natremia and to osmolality in each of the four trials

3a. Crude analysis of favourable neurological prognosis according to natremia category and trial					
Favourable neurological prognosis on day 180, n (%)	All (n = 417/1135)	HYPERION (n = 46/560)	TTM24/48* (n = 221/345)	COMACARE (n = 78/120)	XENON (n = 72/110)
Normonatremia	363/893 (40.7)	40/408 (9.8)	198/295 (67.1)	63/95 (66.3)	62/95 (65.3)
Hyponatremia	50/211 (23.7)	3/125 (2.4)	22/46 (47.8)	15/25 (60.0)	10/15 (66.7)
Hypernatremia	4/31 (12.9)	3/27 (11.1)	1/4 (25.0)	0/0 (0.0)	0/0 (0.0)

3b. Crude analysis of favourable neurological prognosis according to osmolality category and trial					
Favourable neurological prognosis on day 180, n (%)	All (n = 188/730)	HYPERION (n = 42/507)	TTM24/48* (n = –)	COMACARE (n = 74/114)	XENON (n = 72/109)
Normo-osmolality	151/490 (30.8)	24/299 (8.1)	–	62/96 (64.6)	65/95 (68.4)
Hypo-osmolality	12/80 (15.0)	3/65 (4.6)	–	6/9 (66.7)	3/6 (50.0)
Hyperosmolality	25/160 (15.6)	15/143 (10.5)	–	6/9 (66.7)	4/8 (50.0)

*Data not available

**Fig. 2:**
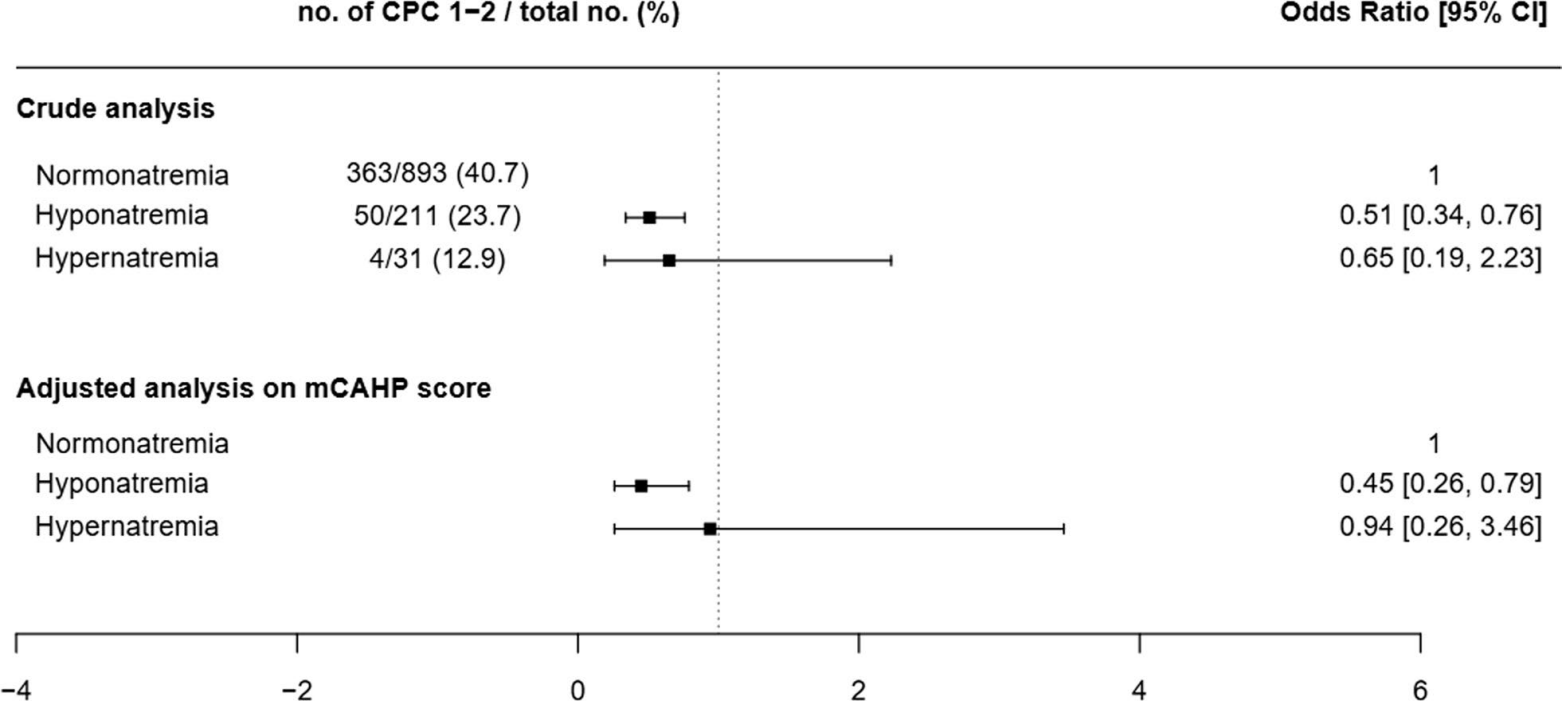
Forest plot of the association between a favourable neurological outcome and the natremia category

By univariate analysis, CPC 1 or 2 on day 180 was significantly less common in the group with hyponatremia than in the group with normal natremia (50/211 vs. 363/893; *p* = 0.001). This association persisted after adjustment on the mCAHP score (adjusted odds ratio [aOR], 0.45; 95% confidence interval [95%CI], 0.26–0.79; *p* = 0.005). Hypernatremia was not significantly associated with day-180 CPC, but the number of patients was very small (CPC 1 or 2, 4/31 vs. 363/893 overall, *p* = 0.5) (Fig. 2). Similar results were obtained when hyponatremia and hypernatremia data from the HYPERION trial were excluded (for hyponatremia: aOR, 0.60 [0.38;0.96]; *p* = 0.03); for hypernatremia: aOR, 0.17 [0.02;1.62]; *p* = 0.12).

Mean osmolality was 287 ± 12 mOsmol·kg^−1^ overall. Hypo-osmolality and hyperosmolality were found in 80/1163 (10.9%) and 160/1163 (21.9%) patients, respectively. In the group with hypo-osmolality, CPC 1 or 2 on day 180 was not significantly more common by univariate analysis than in the group with normal osmolality (OR, 0.64 [0.30–1.39]; *p* = 0.26). This association remained non-significant after adjustment on the mCAHP score (aOR, 0.25; 95%CI 0.20–1.47; *p* = 0.23). Hyperosmolality was not significantly associated with the primary outcome (OR, 1.10 [0.62–1.96], *p* = 0.73 and aOR, 1.34 [0.66–2.74], *p* = 0.42).

### Associations between natremia and day-180 survival

Additional file 1: Table E1 reports the associations between natremia and day-180 survival

## Discussion

In a cohort obtained by pooling the populations of four RCTs that tested neuroprotective interventions after cardiac arrest, natremia early after the ROSC varied widely, with nearly a fifth of patients having hyponatremia and only 3% hypernatremia. Hyponatremia was associated with a lower probability of a favourable functional outcome on day 180, even after adjustment on potential confounders assessed using the mCAHP score. Neither hypo-osmolality nor hyperosmolality was associated with a favourable functional outcome by day 180, suggesting that natremia was the relevant factor.

Abnormalities in serum sodium levels adversely affect pivotal physiological parameters such as intracellular hydration [23] and increase both morbidity and mortality [24]. Thus, in the general ICU population, hyponatremia and hypernatremia were independently associated with increased mortality [8]. One of the few studies of natremia in patients comatose after cardiac arrest and ROSC had a large sample size of 5160 and found that survival with a favourable outcome after 1 month was 17.6%, 8.2%, and 5.7% in the groups with normal, low, and high natremia, respectively [25]. In another retrospective study, early hypernatremia was associated with a lower probability of a favourable CPC after 1 year (aOR for each 1 mmol/L increase, 1.13; 95%CI 1.04–1.23; *p* = 0.004 [26]). In unselected patients admitted to a cardiac ICU, both hyponatremia and hypernatremia on admission were associated with higher in-hospital and 5-year mortality rates, both before and after adjustment [27]. However, these results are merely associations, and whether the brain injury secondary to cardiac arrest contributes to cause and/or is worsened by natremia abnormalities remains to be determined.

Few studies have investigated interventions designed to modify serum sodium levels after cardiac arrest. In an RCT, hypertonic saline infusion (7.2% NaCl) during resuscitation for OHCA did not affect survival but induced a small yet significant increase in the proportion of patients whose CPC was 1 or 2 at hospital discharge (OR, 2.9; 95%CI 1.004–8.5) [28]. The intervention significantly increased serum sodium to a mean of 162 ± 36 mmol/L, but this effect was short-lived, with near-normal levels at ICU admission in the hospital. That hypernatremia was not associated with the functional outcome in our study might indicate either that hypernatremia early after cardiac arrest does not have adverse effects or that hypernatremia is merely a marker for other physiological variables or comorbidities. A matched-pair study indicated that the same strategy significantly increased the proportion of patients who achieved ROSC (OR, 2.19; 95%CI 1.6–3.0). However, in an RCT in patients with traumatic brain injury, a continuous infusion of 20% hypertonic saline failed to improve the day-180 functional outcome [29]. RCTs in patients with post-cardiac-arrest brain injury face major obstacles: no reproducible and effective triage tool is available, the multiple interventions used for resuscitation may affect serum sodium levels, any possible added benefits from altering serum sodium levels may be small and therefore difficult to detect compared to the combined effects of all the other treatments used, and the use of placebos in patients with cardiac arrest raises ethical concerns [30]. Although not recommended in current guidelines outside specific situations, bicarbonate infusion during resuscitation is often used in North America and may act in part via effects on serum sodium levels and not only on metabolic acidosis [31]. In a swine model, hypertonic sodium lactate infusion during or after cardiac-arrest resuscitation increased natremia and plasma osmolarity, improved haemodynamics, and decreased the brain injury marker glial fibrillary acid protein in plasma [32]. The intravenous fluids frequently injected after the ROSC to prevent or treat post-resuscitation shock also affect natremia [33]. In an RCT in 11 052 patients, including 486 with traumatic brain injury, a balanced solution was compared to 0.9% saline (containing about 10% more sodium) [34]. The day-90 mortality rate was slightly but significantly higher in the balanced-solution group (aOR, 1.48; 95%CI 1.03–2.12; *p* = 0.02) In a post hoc analysis of data from the SMART trial in unselected ICU patients, outcomes were compared in the subgroups with traumatic brain injury (698 given balanced crystalloid and 665 given 0.9% saline) [35]. Day-30 mortality was not significantly higher in the balanced-crystalloid group (aOR, 1.03; 95%CI 0.60–1.75; *p* = 0.913), although the risk of either in-hospital death or discharge to another medical facility was significantly increased (aOR, 1.38; 95%CI 1.02–1.86; *p* = 0.04). In addition to intravenous fluid administration, other interventions also affect serum sodium levels. Thus, therapeutic hypothermia at 33 °C is associated with a mild increase in natremia [36]. Finally, in patients with dysnatremia, the speed of natremia correction, and not only the natremia abnormalities themselves, may affect outcomes. Thus, rapid natremia correction was historically associated with osmotic demyelination syndrome in unselected patients admitted to the hospital with hyponatremia, although rapid correction was also associated with better survival in ICU patients [9, 16].

A major limitation of our study is the retrospective design. Although the data were collected prospectively for RCTs, we cannot assess causality links. Moreover, the primary outcome for the current study (day-180 CPC 1 or 2) was the primary outcome in only one of the four trials; it was a secondary outcome in two trials and, for the remaining trial, the primary outcome was the day-90 CPC, which we extrapolated to day 180 for the present study. Second, we did not collect data on dysnatremia correction but, instead, considered only dysnatremia on ICU admission. As indicated above, whether and how rapidly dysnatremia is corrected may affect outcomes. Third, the causes of dysnatremia may also affect outcomes. Thus, some causes of cardiac arrest, as well as comorbidities and chronic treatments common in patients with cardiac arrest, may cause dysnatremia. Also, natremia may be modified by the treatments used for resuscitation and by post-cardiac arrest brain injury. The links between dysnatremia and outcomes are therefore difficult to elucidate. Whether dysnatremia and its correction influence outcomes could only be determined by RCTs. Fourth, the RCTs whose data we used tested different interventions, which may have interacted with any potential effects of dysnatremia. Finally, we handled natremia as a categorical variable and not as a continuous variable. However, categorising natremia as normal or abnormal may be more relevant to bedside care.

## Conclusions

Amongst ICU patients who remain comatose after cardiac arrest followed by ROSC, about a fifth had hyponatremia at ICU admission, whereas hypernatremia was far less common. Hyponatremia was associated with a lower probability of a favourable functional outcome on day 180, even in the adjusted analysis. Further RCTs assessing the effect on outcomes of interventions aimed at modifying natremia in patients with ROSC and persistent coma after cardiac arrest are warranted.

### Supplementary Information

**Additional file 1: eTable 1.** Day-180 survival rates in the four trials according to natremia and plasma osmolality

**Additional file 2: eFigure 1.** Receiver operating characteristic curve for the modified Cardiac Arrest Hospital Prognosis score as a predictor of a favourable functional outcome (Cerebral Performance Category 1 or 2 on day 180).

## Data Availability

The study data will be shared with investigators upon reasonable request to the corresponding author.

## References

[1] Meyfroidt G, Bouzat P, Casaer MP (2022). Management of moderate to severe traumatic brain injury: an update for the intensivist. Intensive Care Med.

[2] Kirkegaard H, Taccone FS, Skrifvars M, Søreide E (2019). Postresuscitation care after out-of-hospital cardiac arrest: clinical update and focus on targeted temperature management. Anesthesiology.

[3] Zeiner A, Holzer M, Sterz F (2001). Hyperthermia after cardiac arrest is associated with an unfavorable neurologic outcome. Arch Intern Med.

[4] Nielsen N, Wetterslev J, Cronberg T (2013). Targeted temperature management at 33°C versus 36°C after cardiac arrest. N Engl J Med.

[5] Lascarrou J-B, Merdji H, Le Gouge A (2019). Targeted temperature management for cardiac arrest with nonshockable rhythm. N Engl J Med.

[6] Dankiewicz J, Cronberg T, Lilja G (2021). Hypothermia versus normothermia after out-of-hospital cardiac arrest. N Engl J Med.

[7] Nolan JP, Sandroni C, Böttiger BW (2021). European resuscitation council and European society of intensive care medicine guidelines 2021: post-resuscitation care. Intensive Care Med.

[8] Funk GC, Lindner G, Druml W (2010). Incidence and prognosis of dysnatremias present on ICU admission. Intensive Care Med.

[9] MacMillan TE, Shin S, Topf J (2023). Osmotic demyelination syndrome in patients hospitalized with hyponatremia. NEJM Evid.

[10] Survival after out-of-hospital cardiac arrest in Europe—Results of the EuReCa TWO study—Resuscitation. https://www.resuscitationjournal.com/article/S0300-9572(20)30046-0/fulltext. Accessed 28 Sep 2021 10.1016/j.resuscitation.2019.12.042 32027980

[11] Jousi M, Skrifvars MB, Nelskylä A (2019). Point-of-care laboratory analyses of intraosseous, arterial and central venous samples during experimental cardiopulmonary resuscitation. Resuscitation.

[12] Nelskylä A, Skrifvars MB, Ångerman S, Nurmi J (2022). Incidence of hyperoxia and factors associated with cerebral oxygenation during cardiopulmonary resuscitation. Resuscitation.

[13] Jakkula P, Reinikainen M, Hastbacka J (2018). Targeting two different levels of both arterial carbon dioxide and arterial oxygen after cardiac arrest and resuscitation: a randomised pilot trial. Intensive Care Med.

[14] Kirkegaard H, Soreide E, de Haas I (2017). Targeted temperature management for 48 vs 24 hours and neurologic outcome after out-of-hospital cardiac arrest: a randomized clinical trial. JAMA.

[15] Laitio R, Hynninen M, Arola O (2016). Effect of inhaled xenon on cerebral white matter damage in comatose survivors of out-of-hospital cardiac arrest: a randomized clinical trial. JAMA.

[16] Darmon M, Pichon M, Schwebel C (2014). Influence of early dysnatremia correction on survival of critically ill patients. Shock Augusta Ga.

[17] Heavens KR, Kenefick RW, Caruso EM (2014). Validation of equations used to predict plasma osmolality in a healthy adult cohort. Am J Clin Nutr.

[18] van Swieten JC, Koudstaal PJ, Visser MC (1988). Interobserver agreement for the assessment of handicap in stroke patients. Stroke.

[19] Haywood K, Whitehead L, Nadkarni VM (2018). COSCA (core outcome set for cardiac arrest) in adults: an advisory statement from the international liaison committee on resuscitation. Resuscitation.

[20] Lascarrou JB, Dumas F, Bougouin W (2022). Differential effect of targeted temperature management between 32°C and 36°C following cardiac arrest according to initial severity of illness: insights from two international data sets. Chest.

[21] Maupain C, Bougouin W, Lamhaut L (2016). The CAHP (Cardiac Arrest Hospital Prognosis) score: A tool for risk stratification after out-of-hospital cardiac arrest. Eur Heart J.

[22] Lascarrou JB, Bougouin W, Chelly J (2023). Prospective comparison of prognostic scores for prediction of outcome after out-of-hospital cardiac arrest: Results of the AfterROSC1 multicentric study. Ann Intensive Care.

[23] Hayman EG, Patel AP, Kimberly WT (2018). Cerebral edema after cardiopulmonary resuscitation: A therapeutic target following cardiac arrest?. Neurocrit Care.

[24] Adrogué HJ, Madias NE (2000). Hyponatremia. N Engl J Med.

[25] Shida H, Matsuyama T, Komukai S (2022). Early prognostic impact of serum sodium level among out-of-hospital cardiac arrest patients: a nationwide multicentre observational study in Japan (the JAAM-OHCA registry). Heart Vessels.

[26] Cho EJ, Lee MS, Kwon WY (2022). Hypernatremia is associated with poor long-term neurological outcomes in out-of-hospital cardiac arrest survivors. Am J Emerg Med.

[27] Breen T, Brueske B, Sidhu MS (2020). Abnormal serum sodium is associated with increased mortality among unselected cardiac intensive care unit patients. J Am Heart Assoc.

[28] Breil M, Krep H, Heister U (2012). Randomised study of hypertonic saline infusion during resuscitation from out-of-hospital cardiac arrest. Resuscitation.

[29] Roquilly A, Asehnoune K, Atlanrea Study Group and the SociétéFrançaised’AnesthésieRéanimation (SFAR) Research Network (2021). Continuous infusion of hypertonic saline vs standard care and 6-month neurological outcomes in patients with traumatic brain injury—reply. JAMA.

[30] Miller FG, Silverman HJ (2004). The ethical relevance of the standard of care in the design of clinical trials. Am J Respir Crit Care Med.

[31] Touron M, Javaudin F, Lebastard Q (2022). Effect of sodium bicarbonate on functional outcome in patients with out-of-hospital cardiac arrest: a post-hoc analysis of a French and North-American dataset. Eur J Emerg Med Off J Eur Soc Emerg Med.

[32] Hypertonic sodium lactate infusion reduces vasopressor requirements and biomarkers of brain and cardiac injury after experimental cardiac arrest—PubMed. https://pubmed.ncbi.nlm.nih.gov/37087454/. Accessed 12 May 2023 10.1186/s13054-023-04454-1 PMC10122448 37087454

[33] Jozwiak M, Bougouin W, Geri G (2020). Post-resuscitation shock: recent advances in pathophysiology and treatment. Ann Intensive Care.

[34] Zampieri FG, Machado FR, Biondi RS (2021). Effect of intravenous fluid treatment with a balanced solution vs 0.9% saline solution on mortality in critically ill patients: the basics randomized clinical trial. JAMA.

[35] Lombardo S, Smith MC, Semler MW (2022). Balanced crystalloid versus saline in adults with traumatic brain injury: secondary analysis of a clinical trial. J Neurotrauma.

[36] Kirkegaard H, Grejs AM, Gudbjerg S (2022). Electrolyte profiles with induced hypothermia: a sub study of a clinical trial evaluating the duration of hypothermia after cardiac arrest. Acta Anaesthesiol Scand.

