# Supplementary material for: Dysnatremia at ICU admission and functional outcome of cardiac arrest: insights from four randomised controlled trials
Source: Crit Care. 2023 Dec 1;27:472. doi: 10.1186/s13054-023-04715-z (PMC10693108; doi:10.1186/s13054-023-04715-z)
Supplement: Supplementary file 1 — Additional file 1: eTable 1. Day-180 survival rates in the four trials according to natremia and plasma osmolality [file 13054_2023_4715_MOESM1_ESM.docx]

**eTable 1: Survival data as n (%)**

1. **Crude analysis of survival according to natraemia status and trial**

| **Day-180 survival** | **All**  **(n=633/1135)** | **HYPERION (n=461/560)** | **TTM24/48 (n=99/345)** | **COMACARE (n=38/120)** | **XENON (n=35/110)** |
| --- | --- | --- | --- | --- | --- |
| Normonatraemia | 426/893 (47.7) | 76/408 (18.6) | 219/295 (74.2) | 66/95  (69.5) | 65/95 (68.4) |
| Hyponatraemia | 69/211  (32.7) | 17/125 (13.6) | 26/46  (56.5) | 16/25 (64.0) | 10/15 (66.7) |
| Hypernatraemia | 7/31  (22.6) | 6/27 (22.2) | 1/4  (25.0) | 0  (0.0) | 0  (0.0) |

1. **Crude analysis of survival according to osmolality and trial**

| **Day-180 survival** | **All**  **(n=242/730)** | **HYPERION (n=90/507)** | **TTM24/48* (n=-)** | **COMACARE (n=77/114)** | **XENON (n=75/109)** |
| --- | --- | --- | --- | --- | --- |
| Normo-osmolality | 184/490 (37.6) | 51/299 (17.1) | - | 65/96  (67.8) | 68/95  (71.6) |
| Hypo-osmolality | 19/80  (23.8) | 10/65  (15.4) | - | 6/9  (66.7) | 3/6  (50.0) |
| Hyperosmolality | 39/160 (24.4) | 29/143 (20.3) | - | 6/9  (66.7) | 4/8  (50.0) |
