# Supplementary figures and images for: Dysnatremia at ICU admission and functional outcome of cardiac arrest: insights from four randomised controlled trials
Source: Crit Care. 2023 Dec 1;27:472. doi: 10.1186/s13054-023-04715-z (PMC10693108; doi:10.1186/s13054-023-04715-z)

## Slide 1
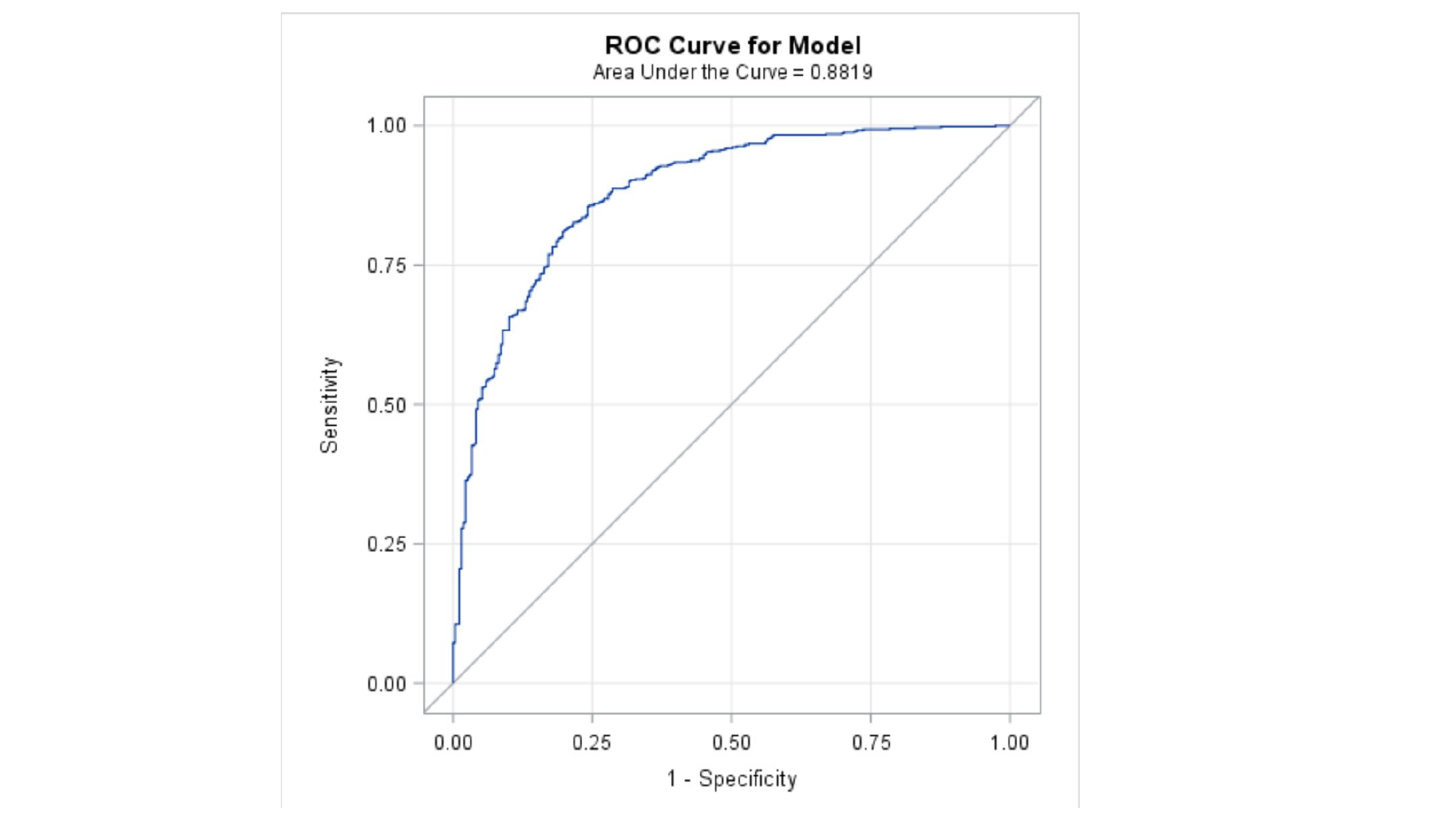

Supplement: Supplementary file 2 — Additional file 2: eFigure 1. Receiver operating characteristic curve for the modified Cardiac Arrest Hospital Prognosis score as a predictor of a favourable functional outcome (Cerebral Performance Category 1 or 2 on day 180). [file 13054_2023_4715_MOESM2_ESM.pptx]
